# Psychological Stressor Of End Stage Chronic Kidney Disease Patients On Dialysis. A Battle For Life

**DOI:** 10.1192/j.eurpsy.2024.1258

**Published:** 2024-08-27

**Authors:** S. S. Hashmi, A. Syed

**Affiliations:** ^1^Department Of Applied Psychology, National University of Modern Languages, Karachi, Pakistan; ^2^First Faculty of Medicine, Charles University, Prague Czechia, Prague, Czech Republic

## Abstract

**Introduction:**

Patients with chronic kidney disease (CKD) may choose to undergo dialysis. Factors that may have led patients to prescribe psychological interventions related to dialysis are poorly understood in the literature. The purpose of this study was to explore multi-level factors surrounding dialysis modalities such as Diagnosed Mood Disorders, Existential crises, Triggering events, Social support, and Distrust towards the process of dialysis.

**Objectives:**

The study aims to investigate the psychological battle of the client while going through the process of dialysis. The study reveals multiple mood disorders and existential crises leading to depression among chronic kidney disease patients. Therefore the study was conducted with the aim of providing a therapeutic guide line in future once the factors are investigated in detail.

**Methods:**

Semi-structured qualitative interviews were conducted in a dialysis clinic in Karachi where 19 participants participated in this qualitative study. The age ranges from 40-76. Initiating with informed consent followed by surveys assessing demographic and clinical information were administered to participants following their interviews.

**Results:**

Qualitative findings suggested that patients were dealing with Clinical Mood Disorders without being provided treatment. Moreover, the cohesive family support enabled them to continue with daily living activities; however, the patients with low support triggering adverse events in life lost their lives in follow-up sessions. Furthermore, nephrology care doesn’t seem sufficient as they are dealing with existential crises of hopelessness, regret, condemnation, and elevated death anxiety. In CKD the misinterpretation of dialysis by cognitively substituting it as End of life increased the clinical symptoms of Mood disorders. Thus the risk factors increase disturbing the quality of life.

**Conclusions:**

Findings point to broader factors affecting dialysis modalities with Mood disorders. The low social support and adverse triggering events precipitate the risk factors of dialysis treatment. Furthermore, distrust towards dialysis and existential crisis are recommended for therapeutic interventions

**Disclosure of Interest:**

None Declared

